# Active Model-Based Hysteresis Compensation and Tracking Control of Pneumatic Artificial Muscle

**DOI:** 10.3390/s22010364

**Published:** 2022-01-04

**Authors:** Yanding Qin, Haoqi Zhang, Xiangyu Wang, Jianda Han

**Affiliations:** 1 Tianjin Key Laboratory of Intelligent Robotics, College of Artificial Intelligence, Nankai University, Tianjin 300350, China; qinyd@nankai.edu.cn (Y.Q.); 2120200408@mail.nankai.edu.cn (H.Z.); wanggxy@mail.nankai.edu.cn (X.W.); 2 Institute of Intelligence Technology and Robotic Systems, Shenzhen Research Institute of Nankai University, Shenzhen 518083, China

**Keywords:** pneumatic artificial muscle, active model, hysteresis compensation, trajectory tracking

## Abstract

The hysteretic nonlinearity of pneumatic artificial muscle (PAM) is the main factor that degrades its tracking accuracy. This paper proposes an efficient hysteresis compensation method based on the active modeling control (AMC). Firstly, the Bouc–Wen model is adopted as the reference model to describe the hysteresis of the PAM. Secondly, the modeling errors are introduced into the reference model, and the unscented Kalman filter is used to estimate the state of the system and the modeling errors. Finally, a hysteresis compensation strategy is designed based on AMC. The compensation performances of the nominal controller with without AMC were experimentally tested on a PAM. The experimental results show that the proposed controller is more robust when tracking different types of trajectories. In the transient, both the overshoot and oscillation can be successfully attenuated, and fast convergence is achieved. In the steady-state, the proposed controller is more robust against external disturbances and measurement noise. The proposed controller is effective and robust in hysteresis compensation, thus improving the tracking performance of the PAM.

## 1. Introduction

Pneumatic artificial muscle (PAM) is a widely-utilized bionic flexible actuator. PAM is mainly composed of a hollow rubber tube, a fiber woven mesh and metal connectors. PAM-driven robots are more flexible than hydraulic or motor-driven robots. In addition, PAM features a higher power-to-mass ratio and good compliance with the human body. Therefore, it has been widely used in the medical and rehabilitation fields. PAM is used in the exoskeleton system, and a terminal sliding mode control was adopted to higher trajectory tracking accuracy [1]. Although PAMs feature good application prospects, the inherent nonlinear hysteresis characteristic has become one of the main obstacles affecting the precise trajectory tracking control of PAM-driven robots. Currently, hysteresis modeling and compensation have gradually become significant areas of research focus [2,3,4,5,6].

From the mathematical point of view, dynamic models for PAMs can be briefly divided into physics-based and phenomenon-based models [7]. The physics-based model mainly analyzes the geometric relationship and material properties of the PAM. For example, C. Kothera et al. considered the thickness of the rubber tube and used the force balance method to derive a static model of a PAM [8]. Because the physics-based model involves very complex mathematical derivation and a large number of model parameters, it creates great difficulties for the design of the controller and adds a very large calculation cost.

Alternately, models based on phenomenology are obtained through experimental data, making the description of the model simpler. For example, in the research work of R. Colbrunn et al., the PAM is regarded as a structure composed of a damping unit, a spring unit and a Coulomb friction unit in parallel [9]. In previous studies, many phenomenological hysteresis models have been proposed and applied to PAMs. The popular hysteresis models can be briefly classified into integral models and differential models. The integral hysteresis model is also called the operator hysteresis model. Common integral hysteresis models include the Maxwell model [10], Preisach model, Krasnosel’skii–Pokrovskii model and Prandtl–Ishlinskii model [11,12]. Differential hysteresis models use nonlinear differential equations to describe hysteresis. Common differential hysteresis models are the Dahl model, LuGre model, Leuven model, Duhem model and Bouc–Wen model [13,14,15,16]. Among them, the Bouc–Wen model can provide acceptable modeling accuracy with fewer parameters. The differential model is attractive in that its formulation can facilitate the modeling, optimization and controller design.

An accurate model is necessary, whereas an increase in the model accuracy often leads to an increase in the model complexity. The model-based closed-loop control strategy can effectively improve the control accuracy. The proportional–integral–derivative (PID) controller is the most widely-used closed-loop controller. Many compensation methods have been proposed to enhance the robustness of PID. For example, T. Nuchkrua proposed a fuzzy self-tuning PID control [17], which generates fuzzy rules based on expert knowledge to adjust the PID gains. T. Thanh combined the traditional PID controller with a neural network, so that the proposed controller featured a strong ability to learn, adapt and deal with nonlinearities [18]. Compared with traditional PID controllers, it is suitable for the control of various objects including linear and non-linear processes. In addition, other control methods have also been proposed for PAMs. For example, W. Zhao designed an extended agent-based sliding mode controller. Experiments verified that the proposed method can effectively reduce jitter [19]. Q. Ai designed a model-free adaptive iterative learning controller. The dynamics of PAM were transformed into a dynamic linearized model in the iterative process to achieve rapid convergence of the tracking errors [20]. Single-neuron adaptive control is also proposed for the hysteresis compensation of nonlinear systems [21].

This paper proposes a hysteresis compensation scheme based on active model control (AMC), where the Bouc–Wen model is used to describe the hysteresis characteristics of the PAM. Firstly, the genetic algorithm is used to identify the Bouc–Wen model parameters. Secondly, the extended state vector is used to actively estimate the system state and modeling error. The unscented Kalman filter (UKF) is used as an estimator for the active estimation [22,23,24]. Ultimately, the active estimator is combined with a nominal controller (the PID controller) to form an actively enhanced PID controller. The experimental results of the nominal controller with and without the AMC verify that the control accuracy can be effectively improved with the help of AMC. Both the transient and steady-state tracking performances have been significantly improved when tracking continuous and noncontinuous trajectories.

The structure of the rest of this paper is arranged as follows. Section 2 introduces the hysteresis characteristics of the PAM and the Bouc–Wen hysteresis model. Section 3 illustrates the active modeling technique. Section 4 shows the architecture of the AMC based control strategy. The experimental results are presented and analyzed in Section 5. The conclusion to this paper is presented in Section 6.

## 2. Bouc–Wen Hysteresis Modeling

### 2.1. Hysteretic Nonlinearity of the PAM

**Figure 1:**
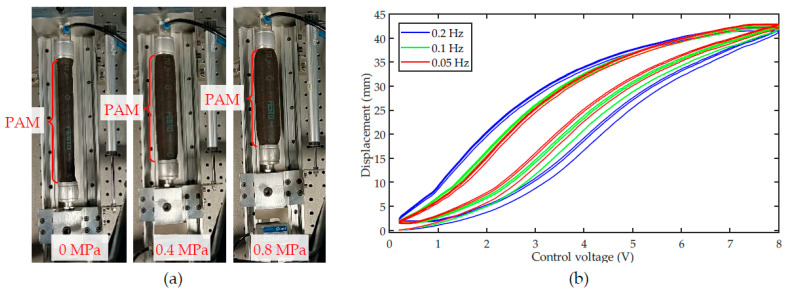
Characteristics of the PAM: (**a**) the contraction of the PAM at different inner pressures, and (**b**) the measured hysteresis loops of the PAM at different frequencies.

PAMs generate contraction displacement and force based on changes in the inner pressure. When the PAM is not pressurized, i.e., the initial state, the length and diameter of the PAM do not change. When the pressure inside the PAM increases, the inner rubber tube produces radial expansion. However, the outer braided mesh hinders the radial movement of the rubber tube, making the length of the PAM shorter in the axial direction, i.e., generating a contraction force. As shown in Figure 1a, with the increment of the inner pressure, the length of the PAM (model DMSP-20-200N from Festo) gradually contracts. When the inner pressure decreases, the PAM gradually returns to its initial state under the action of the outer braided mesh. Due to the friction between the rubber tube and the braided mesh, there is strong hysteretic nonlinearity between the inner pressure and the output displacement and force. 

For instance, for the PAM utilized in Figure 1a, the control voltage to the proportional pressure regulator is adopted as the input and the displacement of the PAM is adopted as the output. Figure 1b shows the measured input–output hysteresis loops of the PAM. It can be observed that the hysteresis loop of the PAM is not symmetric, and the loop shape is influenced by the frequency of the control input, i.e., the rate-dependence. These characteristics increase the difficulty of precisely modeling the hysteresis. 

### 2.2. Bouc–Wen Hysteresis Model

**Figure 2:**
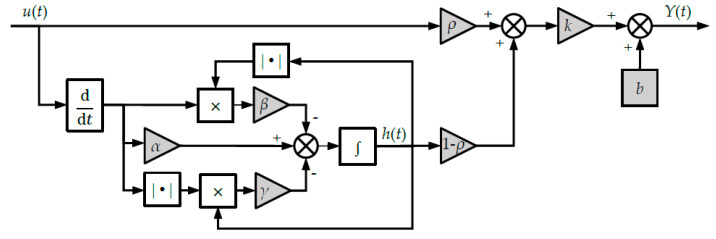
The schematic diagram of the Bouc–Wen model.

In this paper, the Bouc–Wen model is used to describe the hysteresis characteristics of the PAM. The Bouc–Wen model was originally proposed for nonlinear vibration mechanics [25], while currently it is a popular hysteresis model. In the Bouc–Wen model, a state variable *h* is used to characterize the hysteresis relationship of the system. The relationship between the input and the state variable *h* can be described in the following form:

(1) $$\frac{dh}{dt}=\alpha \frac{du}{dt}-\beta \frac{du}{dt}{\left|h\right|}^{m}-\gamma \left|\frac{du}{dt}\right|{\left|h\right|}^{m-1}h$$

where *u* is a generalized input, *h* is the hysteresis state variable, *α*, *β* and *γ* are the gains controlling the shape of the hysteresis loop and *m* controls the smoothness of the transition from elastic to plastic response. In applications, *m* can be set to 1 so as to simplify the model structure. This is also adopted in this paper. For the PAM investigated in this paper, the control voltage applied to the proportional pressure regulator valve is the input of the overall system. As a result, the control voltage is adopted as the input in the Bouc–Wen model. Therefore, the Bouc–Wen model for the PAM can be expressed as follows:

(2) $$\left\{\begin{array}{l} Y(t)=k\rho u(t)+b+k(1-\rho )h(t)\\ \frac{dh}{dt}=\alpha \frac{du}{dt}-\beta \frac{du}{dt}\left|h\right|-\gamma \left|\frac{du}{dt}\right|h \end{array}\right.$$

where *Y*(*t*) is the PAM’s output displacement, *u* is the control voltage, *ρ* ≤ 1 is the weight coefficient, and *k* is the stiffness coefficient of the PAM [26]. The schematic diagram of the hysteresis model is shown in Figure 2, where the parameters of the Bouc–Wen model are shaded in grey background.

## 3. Active Modeling for PAM

In this paper, the Bouc–Wen model defined in (2) is adopted as the reference model in describing the PEA’s hysteresis. Compared with the operator-based hysteresis models, the Bouc–Wen model requires fewer parameters, which is beneficial to the controller design. However, the modeling accuracy of the Bouc–Wen model is limited, especially when it is used to describe the hysteresis characteristics at different pressure ranges or at different frequencies. In order to improve the modeling accuracy, we introduce modeling errors into the reference model. The state and modeling error of the reference model are taken as the extended state vector and a UKF is used to estimate it in real time. Equation (2) can be rewritten as:

(3) $$\left\{\begin{array}{l} Y(t)=k\rho u(t)+b+k(1-\rho )h(t)+V(t)\\ \frac{dh}{dt}=\alpha \frac{du}{dt}-\beta \frac{du}{dt}\left|h\right|-\gamma \left|\frac{du}{dt}\right|h+U(t) \end{array}\right.$$

where *U*(*t*) and *V*(*t*) are the process noise and the observation noise of the system. 

Due to interference and unmodeled uncertainties, there are modeling errors between the actual system and the Bouc–Wen model. We use *f_h_*(*t*) to depict this modeling error, and thus the model of the actual system can be described as:

(4) $$\left\{\begin{array}{l} \dot{\tilde{h}}(t)=\alpha \dot{u}(t)-\beta \dot{u}(t)\left|\tilde{h}(t)\right|-\gamma \left|\dot{u}(t)\right|\tilde{h}(t)+f_{h}(t)+U(t)\\ \tilde{Y}(t)=k\rho u(t)+b+k(1-\rho )\tilde{h}(t)+V(t) \end{array}\right.$$

where $\tilde{h}(t)$ and $\tilde{Y}(t)$ are the state of the actual dynamics of the PAM and the output displacement of the system.

Currently, closed-loop systems often run at a high sampling rate, typically 1 kHz or above. For this high sampling rate, the model error *f_h_*(*t*) can be treated as a slowly changing parameter and it can be approximated as:

(5) $$\begin{array}{l} f_{h}(t)=\dot{\tilde{h}}(t)-\dot{h}(t)\\ {\dot{f}}_{h}(t)=\vec{0}+p(t) \end{array}$$

where *h*(*t*) is the state of the reference model in (2) and *p*(*t*) is assumed to be the process noise actuating the model errors. Subsequently, the discrete description of (4) is obtained:

(6) $$\left\{\begin{array}{l} {\tilde{h}}_{k+1}={\tilde{h}}_{k}+\left[\alpha u_{\delta }{}_{\left(k\right)}-\beta u_{\delta }{}_{\left(k\right)}\left|{\tilde{h}}_{k}\right|-\gamma \left|u_{\delta }{}_{\left(k\right)}\right|{\tilde{h}}_{k}+f_{h}{}_{\left(k\right)}\right]T_{s}+U_{k}\\ f_{h}{}_{\left(k+1\right)}=f_{h}{}_{\left(k\right)}+p_{k}\\ {\tilde{Y}}_{k}=k\rho u_{k}+b+k(1-\rho ){\tilde{h}}_{k}+V_{k} \end{array}\right.$$

where *u_δ_*_(*k*)_ = (*u*_(*k*) −_ *u*_(*k*−1)_)/*T_s_* is the discrete expression of the derivative of the input voltage, *T_s_* is the sample time,${\tilde{h}}_{k}$, *h_k_*, *u_k_*, *f_h(k)_* and ${\tilde{Y}}_{k}$ are the discrete expressions of $\tilde{h}(t)$, *h*(*t*), *u*(*t*), *f_h_*(*t*) and $\tilde{Y}(t)$, respectively and *U_k_* and *V_k_* are the sampling value of *U*(*t*) and *V*(*t*), respectively. 

In this paper, the “joint estimation” technique is adopted [27]. The discrete extended state is defined as follows:

(7) $$X_{k}={\left[{\tilde{h}}_{k}f_{h}{}_{\left(k\right)}\right]}^{T}$$

In order to use UKF, we rewrite (7) as:

(8) $$\begin{array}{c} X_{k+1}=F(X_{k})+U_{k}\\ Y_{k}=H(X_{k})+V_{k} \end{array}$$

where *F* and *H* are the state update function and the measurement function described as:

(9) $$\begin{array}{l} F(X_{k})=\left[\begin{array}{l} F_{{\tilde{h}}_{k}}({\tilde{h}}_{k})\\ F_{f_{k}}(f_{k}) \end{array}\right]=\left[\begin{array}{c} {\tilde{h}}_{k}+[\alpha u_{\delta }{}_{\left(k\right)}-\beta u_{\delta }{}_{\left(k\right)}\left|{\tilde{h}}_{k}\right|-\gamma \left|u_{\delta }{}_{\left(k\right)}\right|{\tilde{h}}_{k}+f_{h}{}_{\left(k\right)}]T_{s}\\ f_{h}{}_{\left(k\right)} \end{array}\right]\\ H(X_{k})=k\rho u_{k}+b+k(1-\rho ){\tilde{h}}_{k} \end{array}$$

Due to the nonlinear characteristics of the Bouc–Wen model in (2), UKF can be used as the estimator. UKF is a well-developed filter widely used for estimation, as in our previous work [22]. Combined with the Bouc–Wen model, UKF includes the following steps. 

The first step is to calculate the sampling points based on the estimated mean and covariance:

(10) $$\chi_{k-1}={\left[X_{k-1}\cdots X_{k-1}\right]}_{n\times (2n+1)}-\left[\begin{array}{ccc} 0_{n\times 1} & -\sqrt{\left(n+\lambda \right)P_{k-1|k-1}} & \sqrt{\left(n+\lambda \right)P_{k-1|k-1}} \end{array}\right]$$

where *n* is the state dimension, *λ* is a constant and *χ_k_*_−1_ is the matrix after sampling expansion, calculating from the estimated mean and covariance calculated on the state vector *X_k_*_−1_.

The second step is the prediction:

(11) $$\begin{array}{cc} \chi_{k|k-1} & =F(\chi_{k-1})\\ P_{X(k|k-1)} & =\sum_{i=1}^{2n+1}\left[W_{c}(i)(\chi_{k|k-1}(i)-W_{m}\chi_{k|k-1})\right.\left.{\left(\chi_{k|k-1}(i)-W_{m}\chi_{k|k-1}\right)}^{T}\right]+Q_{k} \end{array}$$

where *χ_k_*_|*k*−1_ is obtained by the nonlinear conversion of sampling points *χ_k_*_−1_, *Q_k_* is the covariance matrix of process noise and the process noise is added to the covariance prediction step to obtain the weighted covariance matrix in the state space *P_X_*_(*k*|*k*−1)_.

The third step is the update:

(12) $$\begin{array}{cc} \Upsilon_{\chi (k|k-1)} & =H(\chi_{k|k-1})\\ P_{\Upsilon (k|k-1)} & =\sum_{i=0}^{2n}\left[W_{c}(i)(\Upsilon_{\chi (k|k-1)}(i)-W_{m}\Upsilon_{\chi (k|k-1)})\right.\left.{\left(\Upsilon_{\chi (k|k-1)}(i)-W_{m}\Upsilon_{\chi (k|k-1)}\right)}^{T}\right]+R_{k}\\ P_{X\Upsilon (k|k-1)} & =\sum_{i=0}^{2n}\left[W_{c}(i)(\chi_{k|k-1}(i)-W_{m}\chi_{k|k-1})\right.\left.{\left(\Upsilon_{\chi (k|k-1)}(i)-W_{m}\Upsilon_{\chi (k|k-1)}\right)}^{T}\right]\\ K_{k} & =P_{X\Upsilon (k|k-1)}P_{\Upsilon (k|k-1)}^{-1}\\ X_{k|k} & =W_{m}\chi_{k|k-1}+K_{k}(Y_{k}-W_{m}\Upsilon_{\chi (k|k-1)})\\ P_{k|k} & =P_{X\Upsilon (k|k-1)}-K_{k}P_{\chi \Upsilon (k|k-1)}^{T} \end{array}$$

where *R_k_* is the covariance matrix of the measurement noise, $\Upsilon_{\chi (k|k-1)}$ is the measurement prediction of *χ_k_*_−1_ from time *k* − 1 to time *k*, *K_k_* is the Kalman gain, *X_k_*_|*k*_ is the estimation of state vector based on confidence field *χ_k_*_|*k*−1_ and *P_k_*_|*k*_ is the update of confidence matrix *P_k−_*_1*|k−*1_. *W_c_*(*i*) and *W_m_*(*i*) are described below:

(13) $$\begin{array}{cc} W_{m}(0) & =W_{c}(0)=\frac{1}{2(n+\lambda )}\\ W_{m}(i) & =\frac{\lambda }{n+\lambda }\\ W_{c}(i) & =\frac{\lambda }{n+\lambda }+n+\vartheta -\sigma^{2} \end{array}$$

where *λ* = *σ*^2^(*n* + *κ*), *κ* is the scale factor and its value only needs to ensure that the covariance matrix is non-negatively definite, *σ* controls the range of sampling points distribution and the tuning of *ϑ* can improve the approximate accuracy of the covariance. The tuning of the parameters is straightforward. The range of *σ* is 10^−^^4^ ≤ *σ* ≤ 1. A larger *σ* value helps to increase the noise suppression, whereas the robustness might become weaker. As a result, *σ* is usually set to be a smaller value. For Gaussian distribution, *ϑ* can usually be set to 2. Based on related research [23,28], *κ* + *n* = 3, *σ* = 10^−3^ and *ϑ* = 2 are adopted in this paper.

## 4. Active-Model-Based Control Strategy

**Figure 3:**
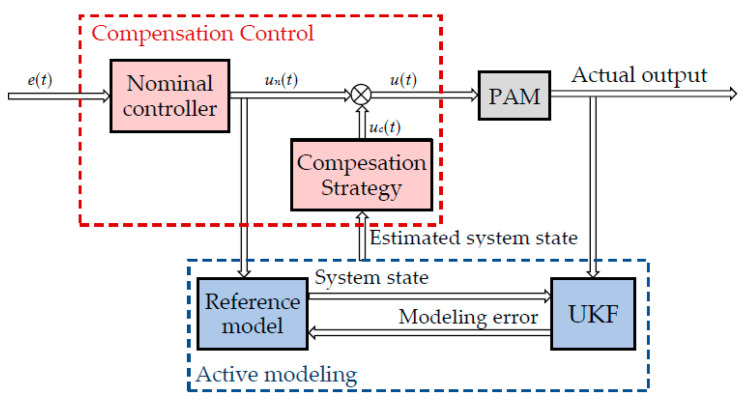
The diagram of the control strategy.

The closed-loop controller proposed in this paper can be divided into two parts. As shown in Figure 3, the first part is the nominal controller. In this paper, the widely-utilized PID controller is selected as the nominal controller. The other part is the state estimation of the system through UKF. For the proposed AMC-based controller, the estimated state of the system is used to compensate the error of the nominal controller via the compensation strategy unit. In this way, the uncertainties of the nominal controller are compensated, and the control performance of the overall system can be improved. In this control strategy, the requirement on the accuracy of the reference model does not need to be too high, because the UKF and compensation strategy will estimate and compensate for the model uncertainty in real time.

The block diagram of the control strategy is shown in Figure 3. The total control voltage of the controller is

(14) $$u_{\left(k\right)}=u_{n}{}_{\left(k\right)}+u_{c}{}_{\left(k\right)}$$

where *u* is the final control voltage applied to the PAM, *u_n_* and *u_c_* are the control voltages generated by the nominal controller and the compensation strategy, respectively. The control objective is expressed as:

(15) $$Y_{d}{}_{\left(k\right)}-{\tilde{Y}}_{k}=0$$

where ${\tilde{Y}}_{k}$ and *Y_d_*_(*k*)_ are the actual and desired trajectories, respectively, and the estimated tracking error is defined as $e_{k}=Y_{d}{}_{\left(k\right)}-{\tilde{Y}}_{k}$. In this paper, the PI controller is adopted as the nominal controller, which can be expressed in the following form:

(16) $$u_{n}{}_{\left(k\right)}=k_{p}e_{k}+k_{i}\sum_{i=0}^{k}e_{i}$$

where *k_p_* and *k_i_* are the proportional and integral gains. 

Based on the control voltage of the nominal controller and the result of the UKF estimation, the estimated value of ${\hat{\tilde{Y}}}_{k+1}$ can be obtained, which can be expressed as follows:

(17) $$\left\{\begin{array}{l} {\hat{\tilde{h}}}_{k+1}={\tilde{h}}_{k}+[\alpha u_{n\delta }{}_{\left(k\right)}+\beta u_{n\delta }{}_{\left(k\right)}\left|{\tilde{h}}_{k}\right|-\gamma \left|u_{n\delta }{}_{\left(k\right)}\right|{\tilde{h}}_{k}+f_{h}{}_{\left(k\right)}]T_{s}\\ {\hat{\tilde{Y}}}_{k+1}=k\rho u_{n}{}_{\left(k\right)}+b+k(1-\rho ){\hat{\tilde{h}}}_{k+1} \end{array}\right.$$

where *u_nδ_*_(*k*)_ = [*u_n_*_(*k*)_ − *u_n_*_(*k*−1)_]/*T_s_* is the discrete expression of the derivative of the nominal control input. The predicted tracking error is defined as ${\hat{e}}_{k+1}=Y_{d}{}_{\left(k+1\right)}-{\hat{\tilde{Y}}}_{k+1}$. In this paper, after getting the prediction error, we define the compensation strategy as follows

(18) $$u_{c}{}_{\left(k\right)}=k_{c}{\hat{e}}_{k+1}$$

where *k_c_* a proportional gain. Thus, the final control input *u* is obtained:

(19) $$u_{k}=k_{p}e_{k}+k_{i}\sum_{i=0}^{k}e_{i}+k_{c}{\hat{e}}_{k+1}$$

## 5. Experimental Results and Analyses

### 5.1. Experimental Setup

**Figure 4:**
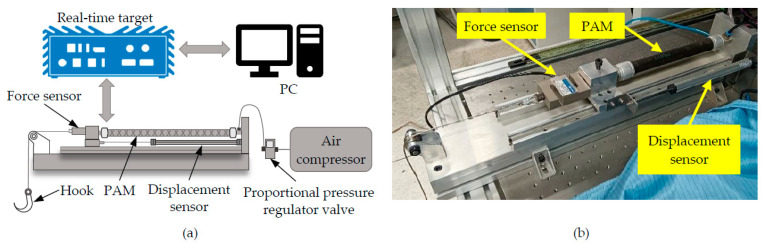
The experimental setup: (**a**) schematic diagram of the overall system and (**b**) the picture of the testbench.

In order to verify the actual performance of the proposed control method, multiple sets of trajectory tracking experiments are implemented on an in-house built testbench for PAMs. The experimental setup is shown in Figure 4, where different PAMs are installed on a linear rail. The Novotechnik displacement sensor is used to measure the displacement of the PAM with an accuracy of 0.001 mm. In this paper, the nominal stroke of the selected PAM (model DMSP-20-200N from Festo) is 40 mm at a maximum pressure of 0.8 MPa. The proportional pressure regulator valve (model VPPM-6L-L from Festo) is used to control the inner pressure of the PAM. The data acquisition and closed-loop control are implemented on a real-time target machine (Model Mobile from Speedgoat) at a sampling rate of 1 kHz.

### 5.2. Identification of the Bouc–Wen Model

As shown in (2), there are six parameters to be identified in the Bouc–Wen model, i.e., *k*, *ρ*, *b*, *α*, *β* and *γ*. Genetic algorithm (GA) is adopted as the identification algorithm. In the open-loop condition, a sinusoidal signal of *u*_0_(*t*) = 2.9 sin (0.2π*t*) + 0.2 is applied to the pressure regulator valve as the control voltage. The displacement of the PAM is measured and used for parameter identification. We use the mean square error (MSE) in the following form as the fitness function for the GA:

(20) $$MSE=\frac{1}{N}\sum_{i=1}^{N}{\left[Y_{actual}(i)-Y_{model}(i)\right]}^{2}$$

where *N* is the number of sampling point, *i* is the sampling index, *Y_actual_* is the measured displacement and *Y_model_* is the Bouc–Wen model output. 

**Figure 5:**
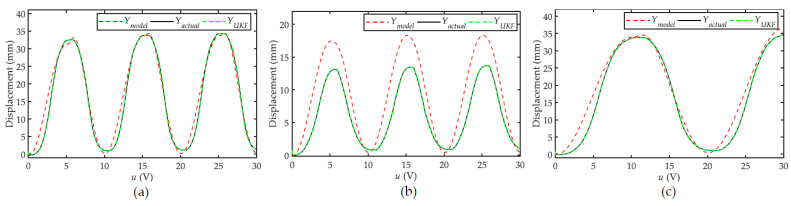
Verifications of the modeling accuracy of the reference hysteresis model with and without active modeling at different frequencies and amplitudes: (**a**) *u*_0_ = 2.9 sin (0.2π*t*) + 0.2, (**b**) *u*_1_(*t*) = 1.4 sin (0.2π*t*) + 0.2, and (**c**) *u*_2_(t) = 2.9 sin (0.1π*t*) + 0.2.

The identified results are *k* = 6.835, *ρ* = 0.987, *b* = −0.569, *α* = −0.188, *β* = 1.106 and *γ* = 0.838. The measured displacement and the model output are plotted in Figure 5a. The model output can match the measurement, whereas the modeling accuracy is not very high.

In order to verify the modeling accuracy of the identified Bouc–Wen model, a further two sinusoidal control signals with different amplitudes and frequencies are used to excite the PAM, i.e., *u*_1_(*t*) = 1.4 sin (0.2π*t*) + 0.2 and *u*_2_(*t*) = 2.9 sin (0.1π*t*) + 0.2. The measured displacements and model outputs are presented in Figure 5b,c. In Figure 5b, the amplitude of *u*_1_ is different from *u*_0_, leading to a significant drop on the modeling accuracy of the Bouc–Wen model. In Figure 5c, the frequency of *u*_2_ is different from *u*_0_. In this case, the modeling accuracy is comparable to *u*_0_. The following root mean square error (RMSE) is used to quantitatively evaluate the modeling errors:

(21) $$\mathrm{RMSE}=\sqrt{\frac{1}{N}\sum_{i=1}^{N}{\left[Y_{model}(i)-Y_{actual}(i)\right]}^{2}}$$

As listed in the first row of Table 1, the RMSE values are calculated to be 2.012 mm, 3.687 mm and 2.282 mm for *u*_0_, *u*_1_ and *u*_2_, respectively.

### 5.3. Active Model Error Estimation

We define the expansion vector in (8) using the actual state of the system and the modeling error. Next, the expansion vector is introduced into the reference model. We use the UKF defined by (9) to estimate the expansion vector in real time, that is, to estimate the actual state and the modeling error of the system at the same time. The modeling accuracy of the active modeling is also investigated and plotted in Figure 5 as a comparison. Compared with the reference model, the system output estimated by the UKF almost coincides with the measured displacement of the system. Similarly, the RMSE is also adopted to evaluate the modeling accuracy of the active model. The RMSE values for *u*_0_(*t*), *u*_1_(*t*), and *u*_2_(*t*) are calculated to be 1.4822 × 10^−4^ mm, 1.379 × 10^−4^ mm and 1.3974 × 10^−4^ mm, respectively. Because the resolution of the displacement sensor is 0.01 mm, such small magnitude errors can be approximated to zero. The RMSE of the active model is also listed in the second row of Table 1. 

Based on the above experimental results, we can conclude that the reference model, i.e., the Bouc–Wen model, can better account for the frequency variation than the amplitude variation. However, it is difficult to maintain high modeling accuracy. By contrast, with the help of the expansion vector, UKF is effective in estimating the modeling errors. The modeling accuracy can be significantly improved using the proposed active model.

### 5.4. Extended State Observer Based Controller for Comparison

The AMC-based PI controller was established in Section IV. In order to test the performance of the proposed controller, trajectory tracking performances with and without active modeling were experimentally compared. For the purpose of comparison, an extended state observer (ESO) was also integrated with the nominal PID controller, i.e., PID + ESO. In order to use the state observer, the state equation of the system is expressed as follows:

(22) $$\left\{\begin{array}{l} {\dot{x}}_{1}=x_{2}\\ {\dot{x}}_{2}=u_{ESO}+\tau \\ y=x_{1} \end{array}\right.$$

where *u_ESO_* is the control input, *y* is the actual measured value of the system, *x*_1_ and *x*_2_ are the state variables of the system and *τ* is treated as a total distribution including the unmodeled nonlinearities and external disturbance. The value *τ* can be extended as an additional state variable, i.e., *x*_3_ = *τ*. The derivative of *x*_3_ is represented by *p*_0_. Equation (22) can be rewritten as:

(23) $$\left\{\begin{array}{l} {\dot{x}}_{1}=x_{2}\\ {\dot{x}}_{2}=u_{ESO}+x_{3}\\ {\dot{x}}_{3}=p_{0}\\ y=x_{1} \end{array}\right.$$

**Figure 6:**
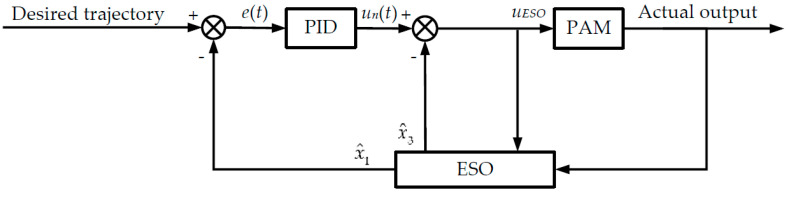
Block diagram of PID + ESO controller.

The extended system model described in (23) is observable. A ESO proposed in [29] is used to estimate system states, which can be constructed as follows:

(24) $$\begin{array}{cc} e & ={\hat{x}}_{1}-x_{1}\\ {\dot{\hat{x}}}_{1} & ={\hat{x}}_{2}-\beta_{1}\cdot e\\ {\dot{\hat{x}}}_{2} & =u_{ESO}+{\hat{x}}_{3}-\beta_{2}\cdot \mathrm{fal}(e,1/2,\delta )\cdot e\\ {\dot{\hat{x}}}_{3} & =-\beta_{3}\cdot \mathrm{fal}(e,1/4,\delta )\cdot e\\ \mathrm{fal}(e,a,\delta ) & =\left\{\begin{array}{l} \frac{e}{\delta^{\left(1-a\right)}},\;\left|e\right|\leq \delta \\ \mathrm{sign}(e){\left|e\right|}^{a},\;\left|e\right|>\delta \end{array}\right. \end{array}$$

where $\hat{x}={\left[{\hat{x}}_{1},{\hat{x}}_{2},{\hat{x}}_{3}\right]}^{T}$ is the state estimate and *β*_1_, *β*_2_, *β*_3_ and *δ* are constants. The ESO defined in (24) is combined with the PID controller, the final control voltage is expressed as:

(25) $$u_{ESO}=u_{n}-{\hat{x}}_{3}$$

where *u_n_* is the control voltage of the nominal PID controller and ${\hat{x}}_{3}$ is the estimated state of the total disturbance of the system. The block diagram of PID + ESO is shown in Figure 6. 

### 5.5. Experimental Results

The triangular trajectory is a widely utilized continuous signal. In this section, a 0.1 Hz, 0.2–30 mm triangular trajectory is adopted as the desired trajectory so as to tune the parameters of the controllers. The gains of the nominal PI controller are tuned to be *k_p_* = 0.1 and *k_i_* = 0.15 for a better balance between the transient and steady-state tracking performances. The following parameters are initialized for the UKF in the active model:

(26) $$P_{0}=\left[\begin{array}{cc} 10 & 0\\ 0 & 10 \end{array}\right],\;Q_{0}=\left[\begin{array}{cc} 10 & 0\\ 0 & 10 \end{array}\right],\;R_{0}=0.001$$

The gains of the compensation strategy are set to *k_p_* = 0.1 and *k_i_* = 0.15, *k_c_* = 0.06. For the PID + ESO, the same nominal PI controller is adopted, and the parameters in ESO are tuned to *β*_1_ = *β*_2_ = *β*_3_ = 100, *δ* = 500.

**Figure 7:**
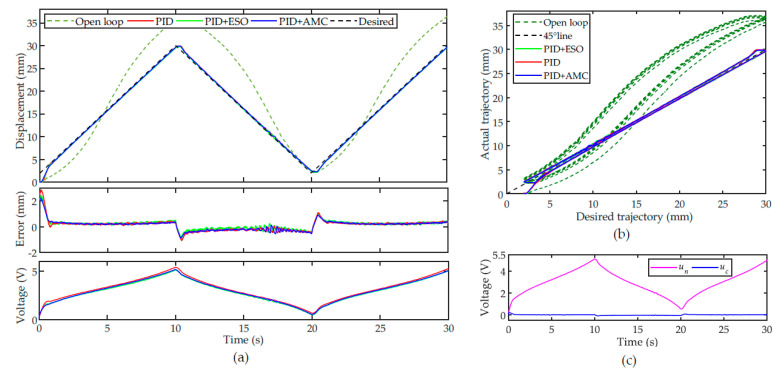
Tracking of a 0.05 Hz triangular trajectory: (**a**) time plot, (**b**) hysteresis compensation performances and (**c**) the control voltages from the nominal controller and the compensation unit.

The tracking performance of the nominal PID controller, PID + AMC and PID + ESO are shown in Figure 7a. For the open-loop system, the tracking performance is poor due to the influence of the hysteresis nonlinearity. For the nominal PI controller, the PAM can follow the desired trajectory well, except for the slight overshoots at the turning points of the trajectory. For the PID + AMC and PID + ESO controllers, the overshoots at the turning points can be suppressed. Because this trajectory is a slow-varying trajectory, the steady-state performances of the controllers are comparable. Similar to the evaluation of the modeling accuracy, the RMSE is utilized to quantitively evaluate the tracking performance of the controllers. The RMSEs of the PID, PID + ESO, and PID + AMC controllers are calculated to be 0.3461 mm, 0.3243 mm and 0.3209 mm, respectively. 

The hysteresis compensation performances are illustrated in Figure 7b. Compared with the open-loop system, the hysteresis loop of the closed-loop system stays close to the 45° line. This demonstrates that all the controllers can successfully compensate the PAM’s hysteresis. Figure 7c shows the control voltages from the nominal controller and the compensation unit. It can be found that the with the help of active modeling and compensation, the control input at the turning points can be compensated. 

Based on the above results, we can conclude that the parameters of all the controller have been well tuned. In order to test the robustness of the controllers, all the parameters are fixed, and tracking experiments of different trajectories are performed. Sinusoidal trajectory with varying amplitude is a very good choice for evaluating the hysteresis compensation performance as it can show both the major and minor loops of the PAM’s hysteresis.

**Figure 8:**
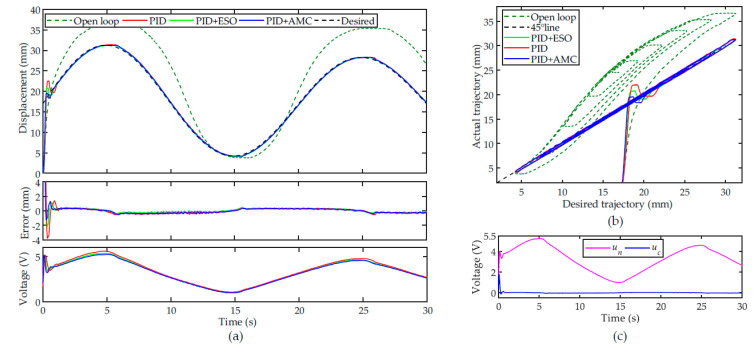
Tracking of a 0.05 Hz sinusoidal trajectory with descending amplitude: (**a**) time plot, (**b**) hysteresis compensation performances and (**c**) the control voltages from the nominal controller and the compensation unit.

The tracking results of a 0.05 Hz sinusoidal trajectory with descending amplitude are shown in Figure 8a. Similar to the performance of the triangular trajectory, the tracking performance of the closed-loop system can be significantly improved by the controllers. However, for the nominal controller, there are obvious overshoot and oscillations in the transient. For the PID + ESO controller, the overshoot and oscillation were successfully suppressed. For the proposed PID + AMC controller, the overshoots and oscillations in the transient were further attenuated. The RMSEs of the PID, PID + ESO, and PID + AMC controllers are calculated to be 0.2450 mm, 0.2005 mm and 0.1934 mm, respectively.

For the hysteresis compensation, as shown in Figure 8b, the nominal controller is efficient at hysteresis compensation, whereas the obvious overshoot and oscillations are not desired in real implementations. It can also be clearly observed in Figure 8b that the proposed PID + AMC controller can improve the transient performance. Figure 8c shows the effect of the active modeling. In the transient, based on the estimated tracking error, the compensation unit can immediately compensate for the nominal controller.

**Figure 9:**
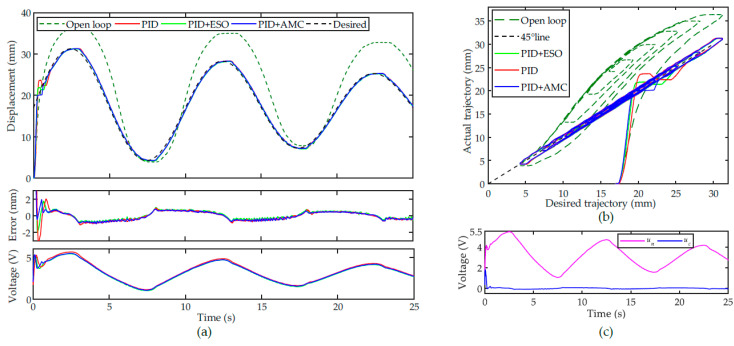
Tracking of a 0.1 Hz sinusoidal trajectory with descending amplitude (**a**) time plot, (**b**) hysteresis compensation performances and (**c**) the control voltages from the nominal controller and the compensation unit.

Experiments were further conducted on tracking another 0.1 Hz sinusoidal trajectory with descending amplitude to test the performances of the controllers for trajectories at different frequencies. The experimental results are provided in Figure 9. Similar to the results in Figure 8, the nominal controller can compensate the hysteresis and the transient tracking performances can be further improved with active modeling. 

The statistics on the tracking errors of the controllers in the above experiments are summarized in Table 2. In each case, the proposed controller can further improve the performance of the nominal controller. 

**Figure 10:**
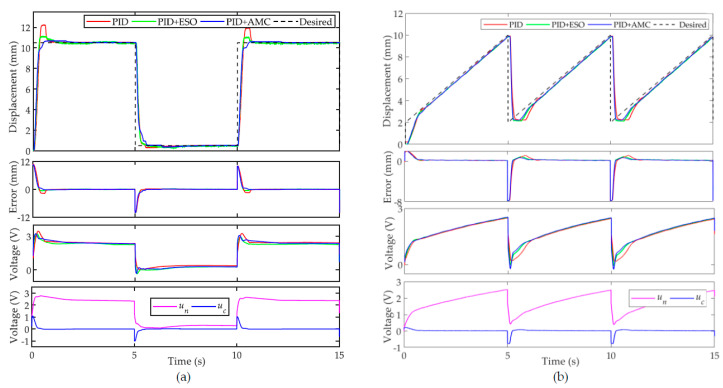
Tracking of discontinuous trajectories: (**a**) square wave trajectory and (**b**) sawtooth trajectory.

It must be pointed out that triangular and sinusoidal trajectories are continuous, i.e., no sudden change occurs in the trajectory. In this case, the hysteresis compensation is relatively simple. In order to test the performance of the controllers in tracking discontinuous trajectories, square wave and sawtooth trajectories are adopted as the desired trajectories. Generally, the controller parameters need to be further tuned for a better performance. In order to test the robustness of the proposed controller, the same controller parameters are inherited. The experimental results are shown in Figure 10. 

Figure 10a shows the tracking performance of the square wave trajectory. For the nominal controller, the transient performance is very poor. For instance, on the forward motion of the PAM, the nominal controller is fast, whereas large overshoot (~17.3%) is observed before the system enters into the steady-state. The settling time was found to be approximately 0.8 s. PID + ESO reduces the overshoot to approximately 5.7%. The proposed PID + AMC can track the square wave signal with negligible overshoot and it can converge within 0.5 s. On the backward motion of the PAM, the performances of all the controllers are comparable, whereas the proposed PID + AMC can still converge quickly without overshoot. The sawtooth trajectory is another popular discontinuous trajectory. As shown in Figure 10b, the nominal controller responds slowly at the turning point of the trajectory. Similar to Figure 10a, the proposed PID + AMC controller offers the best transient performance. For discontinuous trajectories, the sudden change in the trajectory might degrade the transient performance of the nominal controller. It can be seen from the compensation voltage in Figure 10 that the compensation effect of the proposed controller is more obvious at the sudden change of the trajectory.

**Figure 11:**
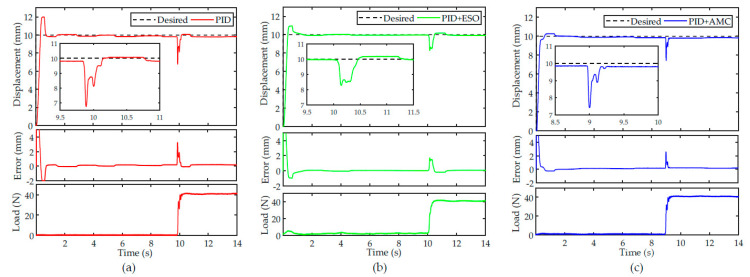
Robustness of the controllers against external disturbances: (**a**) PID, (**b**) PID + ESO and (**c**) PID + AMC. The insets show the zoomed-in details of the transient behaviors of the controllers when confronted with external disturbances.

Further, a payload of approximately 40 N was used to test the robustness of the controllers against external disturbances. It is exerted to the hook when the closed-loop system enters into the steady-state. In this case, it can be regarded as a constant external disturbance. The experimental results are shown in Figure 11. It can be observed that the proposed PID + AMC can quickly converge to its steady-state value, showing excellent disturbance rejection capability. By contrast, it takes longer for PID and PID + ESO to converge. As a result, the proposed controller is more robust against external disturbances.

In the above experimental results, the most important improvement to the proposed controller appears in the transient performance. For the nominal controller, it is difficult to suppress the overshoot and oscillations in tracking different trajectories while maintaining a fast response capability. For the proposed AMC-based controller, due to the high-precision estimation of the modeling error, the tracking performance of the nominal controller can be further improved. During the transient, no obvious overshoot and oscillation exists, and the controller converges faster. We can conclude that high hysteresis compensation efficiency and high robustness are achieved using the proposed controller.

## 6. Conclusions

How to model and compensate the strong hysteresis nonlinearities of the PAM has become one of the main obstacles to the high-precision motion control of PAMs. This paper presents an efficient hysteresis compensation strategy, in which the Bouc–Wen model is adopted as the reference model and a UKF is utilized to estimate the state and the modeling error of the reference model. The estimated error is then utilized as a compensation to the nominal controller so as to improve the performance of the nominal controller. 

Currently, the sampling rate of the closed-loop system can be set high, e.g., 1 kHz or above. In this case, in the steady-state, the estimated tracking error at the next time interval is close to the current tracking error, especially when the PAM is tracking slow and continuous trajectory. However, if the PAM is confronted with a discontinuous trajectory or disturbance, the discrepancy between the estimated tracking error and the current measurement is large. This might lead to poor transient performance. The experimental results demonstrate that for continuous and discontinuous trajectories, the transient performances can be improved by the proposed controller. The proposed controller can successfully eliminate the overshoot and oscillation of the nominal controller and fast convergence is achieved. For the steady-state performance, the proposed controller can better suppress the influence of the measurement noise. Therefore, the effectiveness of the proposed AMC-based hysteresis compensation strategy is verified.

## Figures and Tables

**Table 1:** The RMSE values with and without the active model error estimation. (Unit: mm).

	*u*_0_(*t*)	*u*_1_(*t*)	*u*_2_(*t*)
Reference model	2.012	3.687	2.284
With active model	1.482 × 10^−4^	1.379 × 10^−4^	1.397 × 10^−4^

**Table 2:** The maximum and RMSE of the controllers (Unit: mm).

	Open-Loop (Max/RMSE)	PID (Max/RMSE)	PID + ESO (Max/RMSE)	PID + AMC (Max/RMSE)
0.05 Hz triangular	10.978/6.395	1.097/0.3461	1.100/0.3243	**0.924/0.3209**
0.05 Hz sinusoidal	10.357/6.873	3.769/0.2450	2.322/0.2005	**1.323/0.1934**
0.1 Hz sinusoidal	10.046/6.592	3.272/0.4670	2.010/0.3911	**1.922/0.3803**

## Data Availability

This study did not report any data.
